# Past energy allocation overwhelms current energy stresses in determining energy allocation trade‐offs

**DOI:** 10.1002/ece3.10402

**Published:** 2023-08-08

**Authors:** Blaine D. Griffen, Mikayla Bolander, April Blakeslee, Laura C. Crane, Michele F. Repetto, Carolyn K. Tepolt, Benjamin J. Toscano

**Affiliations:** ^1^ Department of Biology Brigham Young University Provo Utah USA; ^2^ Department of Biology East Carolina University Greenville North Carolina USA; ^3^ Wells National Estuarine Research Reserve Wells Maine USA; ^4^ Department of Biology Temple University Philadelphia Pennsylvania USA; ^5^ Department of Biology Woods Hole Oceanographic Institution Woods Hole Massachusetts USA; ^6^ Department of Biology Trinity College Hartford Connecticut USA

**Keywords:** Asian shore crabs, energetic trade‐offs, *Hemigrapsus sanguineus*, limb regeneration, nonlethal injury, regeneration

## Abstract

Regeneration of lost appendages is a gradual process in many species, spreading energetic costs of regeneration through time. Energy allocated to the regeneration of lost appendages cannot be used for other purposes and, therefore, commonly elicits energetic trade‐offs in biological processes. We used limb loss in the Asian shore crab *Hemigrapsus sanguineus* to compare the strength of energetic trade‐offs resulting from historic limb losses that have been partially regenerated versus current injuries that have not yet been repaired. Consistent with previous studies, we show that limb loss and regeneration results in trade‐offs that reduce reproduction, energy storage, and growth. As may be expected, we show that trade‐offs in these metrics from historic limb losses far outweigh trade‐offs from current limb losses, and correlate directly with the degree of historic limb loss that has been regenerated. As regenerating limbs get closer to their normal size, these historical injuries get harder to detect, despite the continued allocation of additional resources to limb development. Our results demonstrate the importance of and a method for identifying historic appendage losses and of quantifying the amount of regeneration that has already occurred, as opposed to assessing only current injury, to accurately assess the strength of energetic trade‐offs in animals recovering from nonlethal injury.

## Graphical abstract

We show that trade‐offs from previously lost limbs far outweigh trade‐offs from currently missing limbs or limbs that have begun to regenerate. Our results highlight the need to measure historical injury and recovery in order to fully understand energetic trade‐offs in biological systems where nonlethal injury is common.

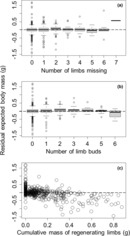

## 1 INTRODUCTION

Energetic constraints are a widespread biological reality, leading to trade‐offs in energy allocation to different biological functions. Life history theory identifies several important trade‐offs, including those between current and future reproduction, growth and reproduction, survival and reproduction, number and size of offspring, and others (Garland et al., 2022; Stearns, 1989). Empirical research over the last several decades provides support for this theory across a wide range of systems. For instance, trade‐offs between current and future reproduction have been documented in leatherback turtles (Rivalan et al., 2005), great tits (Verboven & Tinbergen, 2002), and parasitic wasps (Desouhant et al., 2005). Similarly, growth and reproduction trade‐offs have been shown in kangaroos (Gélin et al., 2016) and mice (Garratt et al., 2020). Further, anole lizards (Cox et al., 2010) and trees (Tuller et al., 2018) have demonstrated trade‐offs between reproduction and survival. Additionally, the trade‐off between the number and size of offspring has been documented across numerous species, including mountain goats (Hamel et al., 2011), humans and other primates (Walker et al., 2008), and marine copepods (Guisande et al., 1996). Trade‐offs, therefore, play a widespread and central role in determining patterns of energy allocation that influence and regulate nearly all aspects of organismal biology.

While trade‐offs occur broadly across taxa, they do not occur indiscriminately in all situations. Instead, they are most acute under stressful conditions (Garland et al., 2022), such as when habitat condition is poor (e.g., stingrays: Dale et al., 2013; owls: Michel et al., 2022) when food quality is low (e.g., reviewed for fish, birds, and mammals: Catoni et al., 2008), or when physical environmental conditions are extreme (e.g., mussels: Petes et al., 2008; frogs: Cramp et al., 2022). Trade‐offs are also common when other aspects of life history or species ecology are energetically demanding, such as in long‐distance migrations (e.g., birds: Buechley et al., 2021; Podlaszczuk et al., 2016) or during molting (e.g., birds: Cornelius et al., 2011; crustaceans: Carvalho & Calado, 2018). This can result in energetic trade‐offs that differ between life history stages because of stage‐specific selection pressures (Schluter et al., 1991).

Nonlethal injury is a common type of energetically demanding stressor that occurs across a wide range of taxa. Examples include dropped limbs in an effort to evade a predator in lizards (Higham et al., 2013) and crustaceans (Juanes & Smith, 1995), as well as partial predation (i.e., predatory consumption of nonessential body parts), such as siphon nipping in bivalves (Lindsay, 2010; Meyer & Byers, 2005) or loss of individual arms in sea stars (e.g., Bingham et al., 2000). In these instances, the nonlethal injury itself may reflect a trade‐off, as dropped limbs may increase survival (Congdon et al., 1974; Emberts et al., 2017) while simultaneously creating an energy deficit because of a decrease in the amount and quality of food consumed following limb loss (e.g., Fazhan et al., 2022; Juanes & Smith, 1995).

Recovery via the regeneration of lost body parts is common in crustaceans (Juanes & Smith, 1995), lizards (Bateman & Fleming, 2009), sea stars (e.g., Bingham et al., 2000), bivalves (e.g., De Vlas, 1985), and other taxa. However, regeneration is energetically demanding and results in additional trade‐offs with growth, reproduction, and survival (reviewed in Bateman & Fleming, 2009; Bely, 2010; Emberts et al., 2019; Fleming et al., 2007; Juanes & Smith, 1995; Maginnis, 2006). Appendage loss in some systems carries additional complexity, in that the lost limb is also a site of energy storage (e.g., the tail in lizards: Price, 2017; the arm in sea stars: Oudejans & Van der Sluis, 1979). Crustaceans, however, store energy primarily in a digestive organ known as the hepatopancreas (Vogt, 2019), thus lost limbs do not result in direct changes to long‐term energy storage. Trade‐offs in crustaceans, therefore, reflect direct costs of limb loss (e.g., reduced amount or quality of food consumed) and regeneration rather than lost energy stores. Despite the energetic impacts of appendage loss, dropping appendages is a common strategy for survival across different taxa. For example, incidence of tail loss in lizards may exceed 50% (Bateman & Fleming, 2009), and limb loss in crustaceans can be as high as 80% in some populations (Juanes & Smith, 1995). The high incidence of appendage loss and its ensuing impacts on growth, reproduction, or survival via trade‐offs means that it has the potential to influence population dynamics.

Current or recent nonlethal injury is easy to detect in most taxa, including crustaceans, as evidenced by missing appendages or appendages that are a fraction of their normal size (i.e., a basal bud) (Hopkins & Das, 2015). However, while limb regeneration begins almost immediately following limb loss, its completion is a gradual process that draws energy throughout the molt cycle (Hopkins, 1993) and can require variable lengths of time to complete. For example, full regeneration of lost limbs can occur in as few as 2–3 molts (e.g., young *Callinectes sapidus*: Smith, 1990; *Menippe mercenaria*: Savage & Sullivan, 1978; *Hemigrapsus edwarsi*: Pringle, 1990), or may take much longer (e.g., 4–7 molts in *Paralithodes camtschatica*: Edwards, 1972). Gradual regeneration likely reflects physiological limitations and/or attempts to regulate trade‐offs by not allocating the total required energy to recovery all at once. As with all biological metrics, there is natural variation in the size (both length and mass) of original limbs for crabs of a given size (e.g., Whittemore et al., 2015). For limb mass, the primary source of this variation is likely time since molting, since body mass increases with soft tissue growth inside the carapace between molts (Mente, 2003). Consequently, as gradually regenerating limbs get closer to their normal size, historical injuries may become harder to visually detect as smaller limb size of mostly regenerated limbs begins to overlap natural variation in the size of original limbs. Yet allocation of resources to continued recovery means that detecting these mostly regenerated appendages and their relative energetic cost is necessary to fully understand trade‐offs associated with recovery from past injuries. For example, experiments performed on the purple shore crab *Hemigrapsus nudus* showed that the number of eggs produced was lowest for crabs that had regenerated previously lost limbs as compared to those that were uninjured or had not yet regenerated lost limbs (Prestholdt et al., 2022). However, this study treated regeneration as an all‐or‐nothing event (yes/no), whereas, in reality, regeneration is a gradual process. Therefore, we hypothesize that the amount of regeneration that has taken place (i.e., the amount of energy that has already been allocated to regeneration) should be inversely proportional to the amount of effort or energy that can be allocated to other processes, including reproduction, growth, and energy storage.

We test this hypothesis using samples collected in a recent study with the Asian shore crab *Hemigrapsus sanguineus* that also looked at energetic trade‐offs associated with limb loss, but that focused only on currently missing limbs that either had not yet begun to regenerate, or where regeneration was nascent (i.e., limb buds were present), but where functioning limbs had not yet been regenerated (Griffen et al., 2022). In this case, limb loss may cause trade‐offs via reduced energy intake or via reallocation of energy to form limb buds. However, this study did not address historic injury or recovery. Using the same specimens that had been used in this previous study, we compared the masses of each specimen's existing limbs relative to body size to determine which limbs were likely in the process of regenerating from historical losses. We then combined recovery from these historical losses with the extent of current injury (missing limbs) and nascent recovery (i.e., limb buds) as predictors in the same statistical models to determine which has the strongest impact on energetics and trade‐offs.

We specifically examine the following hypothesized trade‐offs. First, we hypothesize that energy allocated to limb regeneration will result in altered food consumption. Food consumption could increase with limb loss to meet extra energy demands, but could also decrease with limb loss due to reduced foraging capabilities (Delaney et al., 2011). We expect food consumption to be more strongly influenced by current limb loss than by historical limb loss or regeneration. In addition, we hypothesize that limb loss and regeneration will result in trade‐offs via reduced reproduction (smaller ovary mass), reduced energy storage (smaller hepatopancreas mass), and reduced growth (lower overall body mass), and that these three trade‐offs will be more strongly influenced by energy allocated to regeneration of historically lost limbs than by current injuries, reflecting the energy that has already been allocated to regeneration of historical appendage losses.

## 2 MATERIALS AND METHODS

We used the dataset from Griffen et al. (2022), which included the collection of 799 adult female crabs across five sites along the US East Coast from Maine to North Carolina between March and November 2020. Table 1 provides a summary of collection sites, dates, and sample sizes. A complete description of collections, collection sites, and sample processing is given in Griffen et al. (2022). Briefly, we determined dry weights of ovary, hepatopancreas, cardiac stomach, and the rest of the crab body by removing organs using dorsal dissection and drying each separately to constant weight at 60°C. Sixty‐three of the crabs were missing one of the tissue samples listed above for various reasons and/or experienced limb damage while removing limbs to be weighed (see below) and were discarded from further analysis, leaving 736 crabs for this study. While Griffen et al. (2022) used these same data, that study examined the energetic impacts of injury by noting only the number of missing limbs and the number of newly formed limb buds, whereas this study builds on the previous work and examines the impacts of energy already allocated to limb regeneration.

**TABLE 1:** Crab sampling information.

Site	Latitude, longitude	Julian sampling days of the year (sampling size)
Maine	43°43′2.7336″ N, 70°0′11.4624″ W	75 (20), 133 (30), 192 (41), 252 (34), 314 (32)
New Hampshire	43°2′20″ N, 70°42′55″ W	61 (24), 133 (32), 194 (33), 251 (36), 313 (32)
Connecticut	41°17′56.1″ N, 72°06′44.9″ W	63 (21), 75 (27), 93 (33), 136 (30), 165 (30), 181 (30), 194 (3), 226 (30), 256 (28), 285 (26), 316 (30)
New Jersey	38°58′3.396″ N, 74°57′45.9858″ W	66 (18), 131 (35), 193 (35), 258 (33)
North Carolina	35°46′7.33″ N, 75°31′37.76″ W	75 (27), 136 (30), 197 (5), 259 (14)

*Note*: Information about *Hemigrapsus sanguineus* collected at each of the five sampling sites.

### 2.1 Limb mass as a proxy for energy allocated to limb regeneration

Energy allocated to limb regeneration is evident, in part, as increased metabolism to support protein synthesis of regenerating tissue. Fletcher et al. (2022) measured the metabolic rate of uninjured *H. sanguineus* and of crabs with varying numbers of missing limbs and found that metabolic rate generally increased linearly with the number of limbs that were missing. In addition to this increased metabolic energy loss, energy allocated to regeneration also includes the energy content of the regenerated tissue itself. To verify that this relationship held true in our study, we tested whether limb energy content increases linearly with limb mass.

We extracted the muscle tissue from the first walking leg from 10 different crabs of varying sizes. We weighed the mass of each leg and then combusted each in a Parr 6725 micro‐oxygen bomb calorimeter to determine the energy content. We then used linear regression to determine the relationship between leg energy content and leg mass. A linear relationship, combined with the linear increase in metabolism with number of limbs missing, would support using the mass of regenerated limbs as a proxy for the energy already allocated to limb regeneration.

### 2.2 Detecting historical injury and energy allocated to recovery

Previous work with the congener *Hemigrapsus nudus* showed that limb regeneration is partial at first, requiring multiple molts to regenerate a limb to the size expected if no injury had occurred (Maginnis et al., 2014). This same phenomenon has been shown in numerous other crustaceans and appears to be widespread (reviewed in Juanes & Smith, 1995). We assumed that the same pattern would occur in *H. sanguineus* and that we could detect historical limb losses that were still being regenerated by comparing the observed limb mass to the expected limb mass for a given‐sized crab, had the limb never been lost. While analogous limbs on the two sides of the body are similar in size (*H. sanguineus* is homochelous), the five limbs on one side (e.g., walking leg 2 vs. 3) differ in size and mass. The following procedure was repeated for each of the 10 limbs.

**FIGURE 1:**
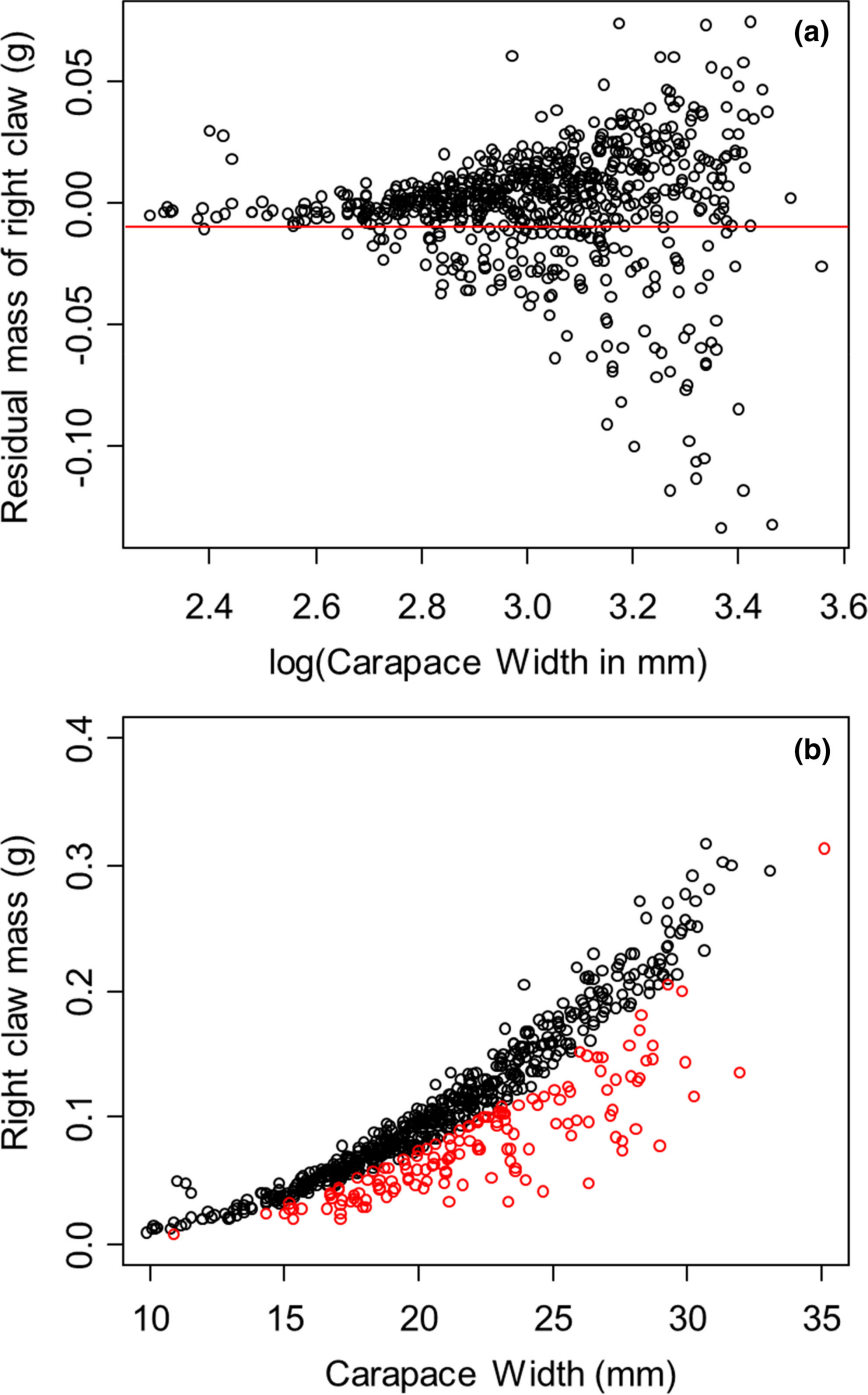
Methods for determining which limbs were regenerating in *Hemigrapsus sanguineus*. Data shown are for the right claw. There is no significance in the choice of this specific limb, as similar relationships were observed for all limbs. (a) Residual limb masses from the nonlinear regression of limb mass on carapace width. Limbs that fall below the red vertical line are assumed to be regenerating. (b) Allometric relationship between limb mass and carapace width. Limbs shown in red are assumed to be regenerating (i.e., these are the limbs that fell below the red line in part a).

For each crab, we removed and independently determined the dry mass of each of the remaining limbs to the nearest 0.01 mg using a Mettler Toledo DualRange scale (model #XS205). For each of the 10 limbs, we first regressed limb mass on carapace width using a nonlinear regression (mass = *a* × carapace width^b^). We took the residuals from that regression and plotted these against log carapace width. For an example of these procedures, see Figure 1. We then visually determined the point where negative residuals broke away from the main cluster of points. For each limb, this generally occurred at residual mass values around −0.01 g (shown by the red horizontal line in Figure 1a). We assumed that anything above this line was a fully developed limb and, therefore, had no evidence that it was regenerating and that anything falling below this line was a regenerating limb. It is possible that this cutoff misrepresents actual regeneration, either by over‐ or underestimating which limbs were being regenerated. Given that variation in limb mass increased with body size (i.e., limb mass is heteroskedastic with carapace width), if errors did occur, they may most likely have resulted in underestimation of regenerating limbs in small crabs and overestimation of regenerating limbs in large crabs. However, the extent of this possible misrepresentation is unclear. In addition, some of these limbs may have been lost and regenerated in the past but had regenerated sufficiently close to their full size by the time of collection so that their loss and regeneration were undetectable using these methods. These methods, therefore, identify only limbs that were not fully regenerated at the time of sampling. Based on these assumptions, the closer a regenerating limb was to this cutoff, the more extensive the regeneration that had already taken place. Thus, the mass of individual regenerating limbs provides an index of the relative energy already invested in regrowing that limb. Lastly, using only the limbs that fell below the cutoff (shown in red in Figure 1b), which were assumed to be regenerating, we summed the mass of regenerating limbs for each individual crab. This total mass of regenerating limbs provides a continuous proxy of the energy that had already been allocated to limb regeneration up to that point. We do not differentiate between individual limbs (e.g., walking legs vs. claws) since all regeneration was combined using the total mass of regenerating limbs.

### 2.3 Relative influence of historical and current nonlethal injury on energetics and trade‐offs

We tested four hypotheses. (1) We tested the hypothesis that food consumption would be influenced by limb loss and the energy that had been allocated to limb regeneration. We did this using the mass of cardiac stomachs (with food inside) as a proxy for the amount of food consumed recently. (2) We tested the hypothesis that reproduction would decrease with limb loss and with increasing allocation of energy to limb regeneration, using the mass of the ovary as a proxy for reproductive effort. (3) We tested the hypothesis that energy storage would decrease with limb loss and with increasing allocation of energy to limb regeneration, using the mass of the hepatopancreas as a proxy for energy storage (Vogt, 2019). (4) We tested the hypothesis that growth would decrease with limb loss and with increasing allocation of energy to limb regeneration, as measured by the total mass of regenerating limbs. Our goal with this fourth hypothesis was to assess growth using changes in body mass; however, body mass may change due to changes in muscle tissue (i.e., growth) or due to changes in ovary or hepatopancreas mass, which we already examined as described above. We, therefore, examined changes in body mass after subtracting out the mass of the ovary and the hepatopancreas. Body mass will also be skewed by the absence of mass from missing limbs. We, therefore, determined the expected mass of each missing limb using the allometric relationship with carapace width for each limb given above and then added this expected limb mass back to the body mass.

We tested each of these four hypotheses using identical statistical approaches but with gut, ovary, hepatopancreas, or body masses (minus the ovary and the hepatopancreas) as response variables, respectively. We tested each hypothesis using a generalized additive model using the mgcv package of R v. 4.2.2 (R Core Team, 2022), with the number of limbs completely missing, the number of limb buds started, and the cumulative mass of the regenerating limbs (not including limb buds) as parametric predictor variables. When graphical analysis suggested that response values varied nonlinearly with predictor variables, we also included squared polynomial terms for the predictors to test for nonlinearity. In these cases, we compared the more complex model to the simplified model without the squared term using AIC to select the best‐fitting model. Limb buds were defined as initiated limbs that were still retained within the cuticular sac (Hopkins & Das, 2015). Previous work using these same samples found a positive correlation between the number of limbs that are missing and the number of limb buds that have been initiated (Griffen et al., 2022). To avoid multicollinearity, we first conducted a generalized linear model with a Poisson distribution to regress the number of limb buds as a function of the number of limbs that were missing (*z* = 27.22, *p* < .0001, null deviance = 1153.83, residual deviance = 545.95). We then used the residual from this analysis (i.e., number of limb buds after accounting for the number of limbs missing) in each of the generalized additive models. Additionally, the amount of food consumed, the ovary mass, the hepatopancreas mass, and the body mass will all increase nonlinearly with body size and may also vary nonlinearly with time of year that the crab was sampled (crabs were sampled from March to November, see Griffen et al., 2022). Our purpose was to control for these nonlinear size‐specific and seasonal changes so that they did not confound the tests of our hypotheses. We, therefore, also included carapace width and Julian sampling date as nonparametric smoothed predictor variables in our generalized additive models. Collection site did not explain any of the variance in our response variables and so was not included in any of the analyses.

In the test of our fourth hypothesis, that there is a trade‐off between limb regeneration and growth, differences between crabs in body mass could reflect changes due to alterations in growth rate (hypothesis tested here), or could simply reflect differences in time since molting, since body mass increases continuously in between molts with soft tissue growth. No methods are available to assess relative time since molting in this species. Unknown differences in time since molting should, therefore, increase the variance in our data, making it more difficult to detect an effect of limb loss and regeneration on growth. However, given our large sample size (*n* = 736), we have confidence in our ability to assess changes in growth despite this increased variance.

**FIGURE 2:**
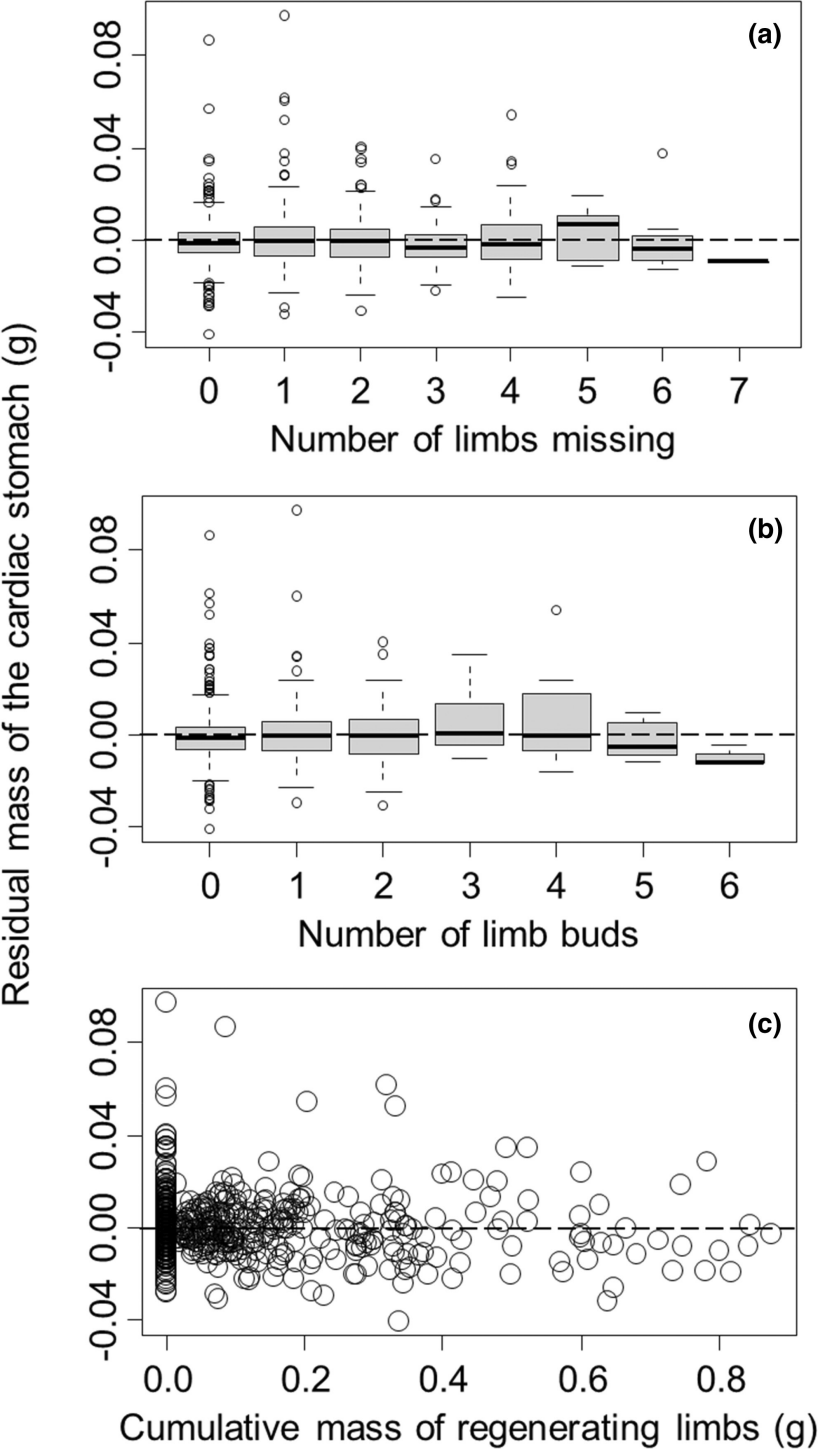
Cardiac stomach mass of *Hemigrapsus sanguineus* (after accounting for body size and day of year) was not significantly influenced by the number of limbs missing (a), showed a unimodal relationship with the number of limb buds started (b), and was only weakly influenced by the mass of historically missing limbs that had already formed (c). Residuals used for visual presentation purposes only. Positive and negative residuals reflect, respectively, stomach masses that are larger or smaller than expected for a given carapace width at that time of year. The dashed horizontal line in each graph highlights the zero point of no change. In each boxplot, the heavy line shows the median, the box encompasses the 1st to 3rd quartile, the whiskers extend to 1.5× this interquartile range, and the circles show outliers that fall outside this range.

**FIGURE 3:**
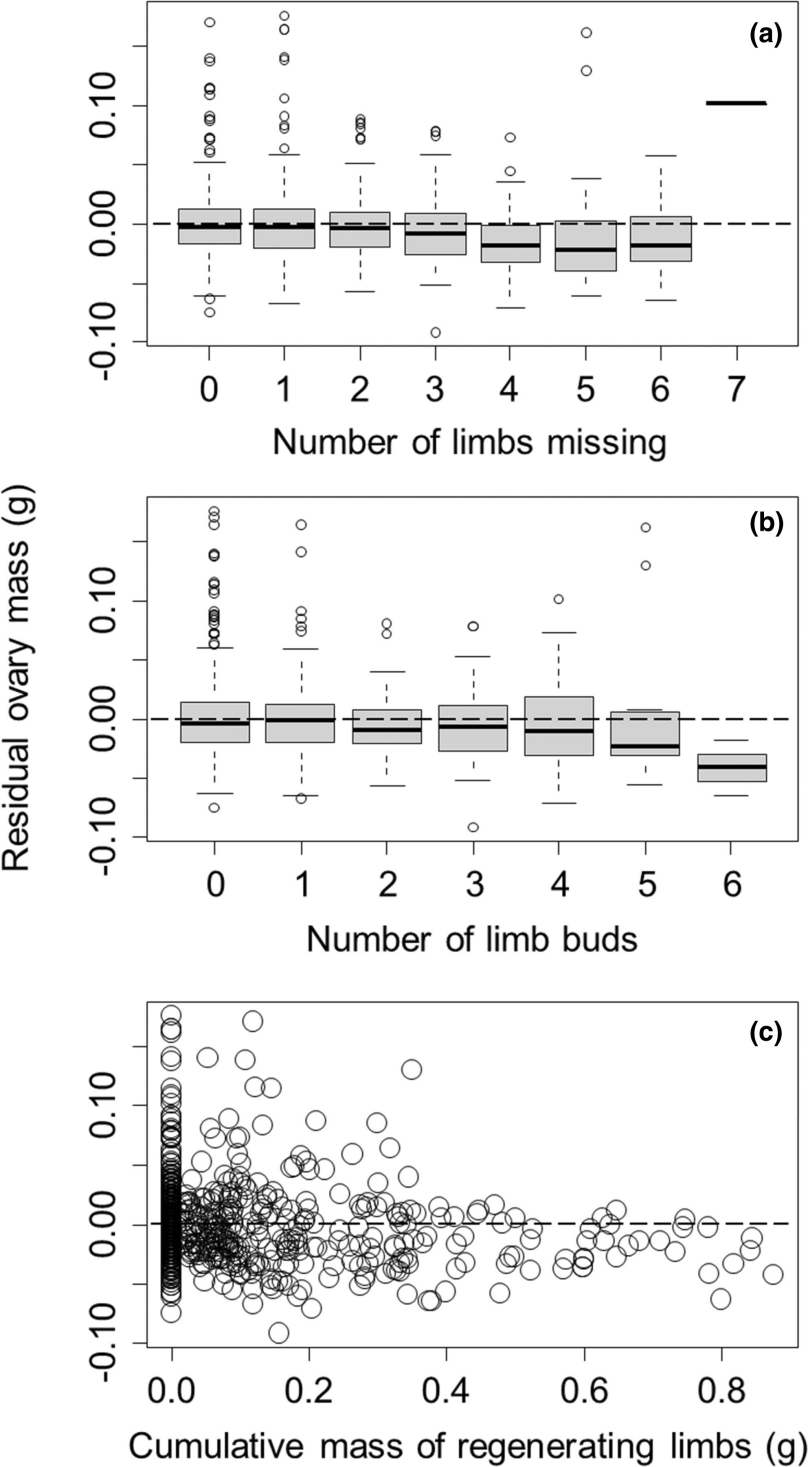
Residual ovary mass of *Hemigrapsus sanguineus* (after accounting for body size and day of year) was not significantly influenced by the number of limbs missing (a), decreased with the number of limb buds that have started (b), and decreased with the cumulative mass of the historically missing limbs that had already formed (c). Residuals used for visual presentation purposes only. Positive and negative residuals reflect, respectively, ovary masses that are larger or smaller than expected for a given carapace width at that time of year. Boxplots and dashed line are as described in the caption to Figure 2.

**FIGURE 4:**
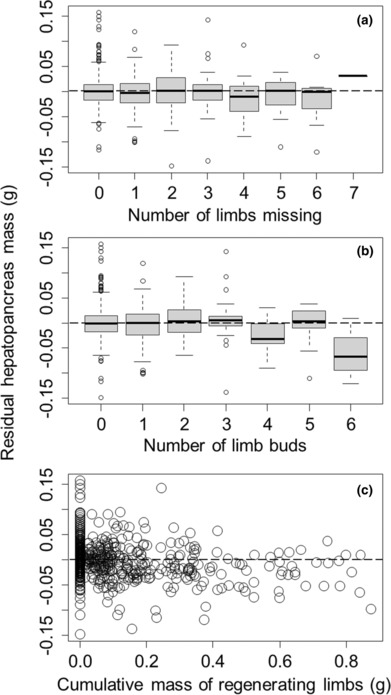
Residual hepatopancreas mass of *Hemigrapsus sanguineus* (after accounting for body size and day of year) decreased with the number of missing limbs (a), and with the number of limb buds that have started (b), and with the cumulative mass of the historically missing limbs that had already formed (c). Residuals used for visual presentation purposes only. Positive and negative residuals reflect, respectively, hepatopancreas masses that are larger or smaller than expected for a given carapace width at that time of year. Boxplots and dashed line are as described in the caption to Figure 2.

**FIGURE 5:**
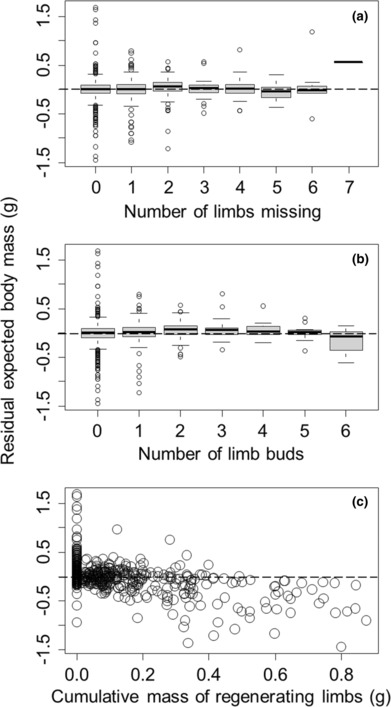
Residual expected body mass (after accounting for body size and day of year) was not significantly influenced by the number of limbs missing (a), but increased with the number of limb buds that have started (b), and decreased with the cumulative mass of the historically missing limbs that had already formed (c). Residuals used for visual presentation purposes only. Positive and negative residuals reflect, respectively, body masses that are larger or smaller than expected for a given carapace width at that time of year. Boxplots and dashed line are as described in the caption to Figure 2.

For graphical illustration purposes only, we have controlled for impacts of body size and time of year in data presented graphically below (Figures 2, 3, 4, 5). We did this for each response variable (gut mass, ovary mass, hepatopancreas mass, or body mass) by fitting these against carapace width and Julian sampling date using a generalized additive model, with both predictor variables smoothed. We then used Pearson residuals from this analysis as a way of graphically showing trends in response variables with limb loss and regeneration without the confounding influence of body size and Julian sampling date.

## 3 RESULTS

### 3.1 Limb mass as a proxy for energy allocated to limb regeneration

Energy content of leg muscle tissue increased linearly with mass, supporting the use of the mass of regenerated limbs as a proxy for the energy allocated to limb regeneration. Specifically, energy content of the limb muscle increased by 21.65 ± 1.38 kJ for each additional gram of dry mass (*t* = 15.74, *p* < .0001, *R* ^2^ = .97).

### 3.2 Detecting historical injury and energy allocated to recovery

The method used here for detecting historical injury cannot detect injury where recovery has been completed. As a result, the patterns shown here are conservative with respect to energetic impacts of historical injury. We found a total of 721 limbs that were missing, representing 9.8% of limbs overall. Of these, 512 had recently started to regenerate, as evidenced by the presence of limb buds. In addition to these recent losses and initial efforts of regeneration, a total of 1046 limbs (~15.8% of existing limbs) were regenerating from having been previously lost. Together, this represents 21.2% of limbs that were either missing or regenerating. Across all crabs, there were a total of 1.49 ± 2.69 (mean ± SD) regenerating limbs detected per crab (range: 0 to 10 limbs), comprising a total dry mass of 89.1 ± 164.6 mg of regenerated tissue per crab (range: 0–876.5 mg). These results are summarized for each limb in Table 2.

**TABLE 2:** For each limb (#1–10), total number of limbs that were either missing, early‐stage regeneration (presence of a limb bud) or substantial regeneration (historically missing).

Limb no.	No. missing	No. missing with limb buds	No. historically missing
1	64	70	93
2	58	35	85
3	82	58	98
4	77	53	107
5	53	28	120
6	6	47	65
7	88	69	129
8	100	68	112
9	70	51	91
10	60	33	146

*Note*: Limbs were numbered beginning with the right claw (1) and numbering incrementally clockwise, ending with the left claw (10).

### 3.3 Relative influence of historical and current nonlethal injury on energetics and trade‐offs

We found that food consumption, using cardiac stomach mass as a proxy, increased nonlinearly with carapace width (*F* = 313.26, *p* < .0001) and varied cyclically with the Julian date on which crabs were collected (*F* = 2.69, *p* = .007) when both were treated as smoothed nonparametric terms in the generalized additive model. After these nonlinearities were accounted for, recent food consumption was not associated with the number of limbs that were missing (*t* = −1.02, *p* = .31, Figure 2a), showed a unimodal relationship with the number of limb buds that had started to form (linear term: *t* = 2.18, *p* = .030; squared term: *t* = 2.97, *p* = .003, Figure 2b), and declined with the cumulative mass of the limbs that had been regenerated (*t* = −2.02, *p* = .043, Figure 2c). ΔAIC of the simpler model (without the squared term) was 6.94. Overall, this model explained 72% of the deviance in cardiac stomach mass.

We found that ovary mass increased nonlinearly with carapace width (*F* = 84.27, *p* < .0001) and varied with the Julian date on which crabs were collected, peaking in summer months (*F* = 28.49, *p* < .0001). After these nonlinearities were accounted for by including them as smoothed nonparametric terms in the generalized additive model, ovary mass was not associated with either the number of limbs currently missing (*t* = −1.44, *p* = .15, Figure 3a), but decreased with the residual number of new limb buds that had formed (*t* = −1.99, *p* = .047, Figure 3b) and decreased by 53.11 ± 9.54 mg for each additional gram of historically lost limbs that had been regenerated (*t* = −5.57, *p* < .0001, Figure 3c). Overall, this model explained 51.1% of the deviance in the ovary mass.

We found that hepatopancreas mass mirrored patterns in gut mass. Specifically, the mass of the hepatopancreas (a proxy for the amount of energy storage) increased nonlinearly with carapace width (*F* = 228.02, *p* < .0001) and varied cyclically with the Julian date on which crabs were collected (*F* = 5.72, *p* < .0001). When these two terms were treated as smoothed nonparametric terms in the generalized additive model, the hepatopancreas mass decreased by 4.88 ± 1.28 mg with each additional limb that was currently missing (*t* = −3.83, *p* = .0001, Figure 4a) and decreased nonlinearly with the number of new limb buds formed (linear term: *t* = 1.76, *p* = .080; squared term: *t* = 2.77, *p* = .006, Figure 4b). Additionally, the mass of historically lost limbs that had been regenerated had a strong impact on energy storage, reducing hepatopancreas mass by 73.76 ± 10.16 mg for each additional gram of limb mass that had been regenerated (*t* = −7.26, *p* < .0001, Figure 4c). ΔAIC of the simpler model without the squared term was 5.35. Overall, this model explained 69.2% of the deviance in hepatopancreas mass.

Consistent with reproduction and energy storage, growth of muscle mass increased nonlinearly with carapace width (*F* = 502.83, *p* < .0001) and varied cyclically with the Julian date on which crabs were collected (*F* = 1.87, *p* = .064) when these terms were treated as smoothed nonparametric terms in the generalized additive model. In addition, growth of muscle tissue was not influenced by the number of limbs that were currently missing (*t* = −1.61, *p* = .11, Figure 5a), but actually increased by 36.62 ± 17.46 mg for each additional new limb bud that had been formed (*t* = 2.10, *p* = .037, Figure 5b). Lastly, regeneration of historically lost limbs had a strong detrimental impact on growth of muscle tissue, reducing muscle growth by 1.24 ± 0.09 g for each additional gram of historically lost limbs that had been regenerated (*t* = −13.95, *p* < .0001, Figure 5c). Overall, this model explained 93.3% of the deviance in residual expected body mass.

## 4 DISCUSSION

We have shown that historical limb loss and regeneration influence the mass of food found in a crab's gut, and that the mass of the ovary (i.e., reproductive effort), the mass of the hepatopancreas (i.e., energy storage), and the mass of the body (i.e., growth) all decrease as the amount of regeneration of lost limbs increases. Further, while energy storage and growth were also, respectively, influenced by the number of currently missing limbs and the number of newly formed limb buds, in each case, the impacts of energy already allocated to limb regeneration was much greater based on parameter estimates of fitted models. These results support our hypothesis that the strength of trade‐offs following appendage loss are in direct proportion to the energy already invested towards regeneration. This finding highlights the importance of detecting historical injuries and quantifying the relative energy already allocated to recovery from these injuries to fully understand energetic trade‐offs associated with the injury and regeneration process.

Our methods for detecting regenerated limbs provide a quantitative approach to assessing the extent of regeneration effort that has already taken place across multiple appendages, and therefore, the physiological costs and trade‐offs associated with limb regeneration following nonlethal injury. These methods can be applied across other crustacean species, as well as to other phylogenetic groups where recovery is gradual (e.g., reptiles, sea stars, mollusks). Our method of using the mass of regenerated tissue is similar to the approach taken by Hodgson (1982) on bivalve siphons; however, most previous studies have focused on length of regenerated limbs (e.g., lizards: Baranowitz et al., 1979; sea stars: Schram et al., 2011; crabs: Maginnis et al., 2014). Our methods for assessing regenerated limb mass were developed after crabs had already been dissected and dried. We, therefore, did not include the mass of new limb buds in our regeneration estimate, and as a result, our estimates are slight underestimates of total regenerated mass. Future studies could include the mass of limb buds to increase accuracy. Overall, we encourage the use of regenerated tissue mass as a way to clearly connect gradual regeneration to costs via changes in the mass of other organs, as we have done here.

We found that the mass of food in the cardiac stomach at the time of capture increased nonlinearly with the number of new limb buds that were formed, but was not influenced by the current number of missing limbs. Increased food consumption with the number of newly formed limb buds could reflect an effort to increase intake to meet the initial energetic requirements of tissue regeneration (Cheng & Yilmaz, 2021). Lack of a correlation between the mass of food found in the gut at any given point in time and current limb loss could reflect the weakness of stomach mass as a proxy for food consumption, as stomach mass alone ignores differences in digestive time and food quality. In addition, variation in the amount of consumption at any given point in time is likely to be strongly influenced by factors other than limb loss, such as food availability across sites and seasons (e.g., Reese et al., 2023). The previous study using these same samples reported that gut mass did increase with the number of missing limbs (Griffen et al., 2022); however, this was not the case after we accounted for crab size and sampling date. Diet quality differs considerably throughout the foraging season for *H. sanguineus*, being highest during the active summer foraging months within the invaded range (Reese et al., 2023), while there is no difference in the prevalence of limb loss with Julian sampling date throughout the invaded range (Griffen et al., 2022). Thus, diet quality appears to be more important than injury in determining the amount of food consumed.

We found that regeneration of previously lost limbs caused a decrease in growth and that this trade‐off had more than a 1‐to‐1 impact on growth. Specifically, we found that for each 1 g of limb mass that had been regenerated, body mass decreased by 1.24 ± 0.09 g, suggesting that this reflects more than simply a shift in energy allocation. The additional lost growth may potentially reflect a shortened molt interval, which is induced by limb loss in some crabs, including in the congener *Hemigrapsus edwardsi* (Pringle, 1990). Crabs that molt early molt at a lower body mass for a given carapace width. This could, therefore, account for the lower body mass than expected for a given size crab that had historically lost limbs. However, another congener, *Hemigrapsus oregonensis*, experiences reduced molt increment (i.e., lower increase in carapace width) in the molt following limb loss (Kuris & Mager, 1975), which would tend to have the opposite effect of early molting by increasing the body mass for a given size crab that had historically lost limbs. Effects of limb loss on molt increment and molt interval in *H. sanguineus* are unknown.

An alternative, though not mutually exclusive, explanation is that the additional lost growth in crabs that had historically lost limbs reflects increased metabolic rates associated with limb regeneration, reflecting the energetic cost of protein synthesis (Hopkins & Das, 2015). The energetic cost of protein synthesis has not been measured in *H. sanguineus* but has been measured in the European green crab *Carcinus maenas* to be 7150 J for each 1 g of tissue synthesized (Houlihan et al., 1990). Protein synthesis should occur both with limb regeneration and with general body growth. The lack of a one‐to‐one trade‐off between growth and regeneration, therefore, suggests increased relative costs of regeneration. And indeed, in *H. sanguineus*, metabolic rate generally increases linearly with the number of limbs that are missing (Fletcher et al., 2022), supporting this conclusion. Similar increases in metabolism following limb loss have been shown in other species (Florida stone crabs: Hancock & Griffen, 2017; flatback marsh crab: Smith et al., 2022).

We found that energy storage in the hepatopancreas decreased with the mass of previously lost limbs that had been regenerated. This finding is in contrast to previous work showing that limb loss itself in this species does not influence energy storage (Griffen et al., 2022). Reduced energy storage with historical limb loss and regeneration may also be explained by both of the arguments presented in the two previous paragraphs. Specifically, if limb loss induces early molting and if molting is energetically costly, then limb loss could reduce energy stores. Additionally, if metabolic rates increase following limb loss to meet the costs of increased protein synthesis (Fletcher et al., 2022), increased costs of locomotion (Escalante et al., 2021), or increased costs of foraging (Hancock & Griffen, 2017), this, combined with reduced food intake that can occur following limb loss (Delaney et al., 2011; Juanes & Hartwick, 1990), can result in an energy imbalance that reduces energy storage.

We found that regeneration of previously lost limbs not only influenced energetic trade‐offs but was the dominant factor in these trade‐offs, overshadowing the impacts of current limb loss. Overall, we found that the trade‐off with growth was the strongest, being 25× stronger and 18× stronger than the trade‐offs with reproduction and energy storage, respectively, while the trade‐off with energy storage was approximately 40% stronger than the trade‐off with reproduction. These differences likely reflect the relative fitness consequences of these trade‐offs and suggest that when pooling samples across all body sizes and sampling times together, individuals facing energy shortages are most likely to reduce growth, followed by reducing energy storage, and are least likely to reduce reproductive effort. While our results suggest that trade‐offs with reproduction are avoided, clearly regeneration has negative impacts on reproduction in this (Griffen et al., 2022) and other systems. It has been suggested that energetic costs of regeneration that force trade‐offs with reproduction may have been responsible for the loss of regenerative capabilities in evolutionary lineages such as mammals and birds (Prestholdt et al., 2022). Previous reviews of autotomy impacts clarify that while such trade‐offs are common, they are highly context‐dependent (Emberts et al., 2019; Fleming et al., 2007; Juanes & Smith, 1995; Maginnis, 2006). To date, we lack a general theoretical framework to make sense of this context dependency and to predict when specific types of trade‐offs with limb regeneration should be expected.

In conclusion, studies that examine trade‐offs following autotomy while ignoring historical limb losses and energy already allocated to regeneration of these lost limbs may provide a skewed picture of overall energetic trade‐offs and are likely to underestimate the true cost of autotomy. The methods used here to detect historic appendage loss and partial regeneration can be applied across a wide range of systems where regeneration happens gradually. Accounting for historic appendage loss in future autotomy studies will provide a more accurate and rigorous understanding of the trade‐offs associated with appendage loss and regeneration.

## AUTHOR CONTRIBUTIONS

**Blaine D. Griffen:** Conceptualization (lead); data curation (supporting); formal analysis (lead); methodology (lead); project administration (lead); writing – original draft (lead). **Mikayla Bolander:** Data curation (equal); writing – review and editing (equal). **April Blakeslee:** Data curation (equal); investigation (equal); writing – review and editing (equal). **Laura C. Crane:** Data curation (equal); investigation (equal); writing – review and editing (equal). **Michele F. Repetto:** Data curation (equal); investigation (equal); writing – review and editing (equal). **Carolyn K. Tepolt:** Data curation (equal); investigation (equal); writing – review and editing (equal). **Benjamin J. Toscano:** Data curation (equal); investigation (equal); writing – review and editing (equal).

## CONFLICT OF INTEREST STATEMENT

The authors have no conflicts of interest to declare.

## Data Availability

All data used in this manuscript are available on Dryad Digital Repository at https://doi.org/10.5061/dryad.6m905qg5h.
